# Degradomics-Based Analysis of Tetanus Toxoids as a Quality Control Assay

**DOI:** 10.3390/vaccines8040712

**Published:** 2020-12-01

**Authors:** Thomas J. M. Michiels, Wichard Tilstra, Martin R. J. Hamzink, Justin W. de Ridder, Maarten Danial, Hugo D. Meiring, Gideon F. A. Kersten, Wim Jiskoot, Bernard Metz

**Affiliations:** 1 Leiden Academic Centre for Drug Research (LACDR), Division of BioTherapeutics, Leiden University, 2333 CC Leiden, The Netherlands; w.jiskoot@lacdr.leidenuniv.nl; 2 Intravacc, Institute for Translational Vaccinology, 3721 MA Bilthoven, The Netherlands; wichard.tilstra@intravacc.nl (W.T.); martin.hamzink@intravacc.nl (M.R.J.H.); justin.de.ridder@intravacc.nl (J.W.d.R.); Maarten.Danial@intravacc.nl (M.D.); hugo.meiring@intravacc.nl (H.D.M.); bernard.metz@intravacc.nl (B.M.)

**Keywords:** degradomics, mass spectrometry, tetanus toxoid, vaccines, proteomics, 3Rs, quality control

## Abstract

Currently, batch release of toxoid vaccines, such as diphtheria and tetanus toxoid, requires animal tests to confirm safety and immunogenicity. Efforts are being made to replace these tests with in vitro assays in a consistency approach. Limitations of current in vitro assays include the need for reference antigens and most are only applicable to drug substance, not to the aluminum adjuvant-containing and often multivalent drug product. To overcome these issues, a new assay was developed based on mimicking the proteolytic degradation processes in antigen-presenting cells with recombinant cathepsin S, followed by absolute quantification of the formed peptides by liquid chromatography-mass spectrometry. Temperature-exposed tetanus toxoids from several manufacturers were used as aberrant samples and could easily be distinguished from the untreated controls by using the newly developed degradomics assay. Consistency of various batches of a single manufacturer could also be determined. Moreover, the assay was shown to be applicable to Al(OH)_3_ and AlPO_4_-adsorbed tetanus toxoids. Overall, the assay shows potential for use in both stability studies and as an alternative for in vivo potency studies by showing batch-to-batch consistency of bulk toxoids as well as for aluminum-containing vaccines.

## 1. Introduction

Tetanus is a severe disease caused by *Clostridium tetani*. Since their development in 1924, tetanus toxoid (TTd) vaccines have dramatically reduced the incidence and the case fatality rate of tetanus [1]. Because tetanus is transmitted by *C. tetani* spores from dirt or soil infecting wounds and not through infected patients, vaccination will always remain an important tool to avoid resurgence of the disease. Similar to the first TTd vaccines [2], the vaccines used today are still based on the principle of inactivating tetanus toxin with formaldehyde. Although these vaccine products are not as pure or well defined as other biologicals (e.g., recombinant insulin or therapeutic antibodies) [3,4,5], they have been used for decades and are safe and efficacious. Currently, in vivo tests are required for batch release to confirm safety (i.e., effective detoxification) and immunogenicity [3,6]. Efforts are being made to implement a consistency approach, where manufacturers that can consistently produce the drug product would only have to prove the potency of a limited number of batches using an animal model and verify consistency of every subsequent batch with in vitro assays [7,8,9]. This could significantly reduce the number of animals needed to verify the potency of toxoid vaccine batches.

In search of a new animal-free assay to confirm the quality of a TTd batch, we looked into the adaptive immune system for inspiration. An important aspect of the adaptive immune system is cellular immunity, in which antigen processing by antigen presenting cells (APCs) plays an important role. After the antigen has been taken up by APCs, proteases in the lysosomes cleave the antigenic proteins and form peptides which are eventually presented by MHC molecules to T-cells. A correlation between slower antigen processing and a stronger immune response suggests that the antigen processing kinetics could be used as a metric for immunogenicity [10,11,12,13,14,15,16]. We hypothesized that by mimicking proteolytic antigen degradation in vitro with the lysosomal endoprotease cathepsin S, the obtained degradation kinetics have the potential to verify the quality of the TTd. A previous study on model proteins showed that differences in formaldehyde-inactivation could be picked up in such an assay [17].

Our aim was to develop a new and reliable mass spectrometry based in vitro assay to show TTd production consistency and (bulk) product stability by measuring cathepsin S degradation. The main advantage of such an assay over other in vitro assays is that it does not need a biological reference but can use well defined synthetic peptides as a standard. To verify that our test could identify aberrant products, heat exposed plain tetanus toxoids (drug substance) as well as control batches of drug substance from various manufacturers were analyzed and the results of the degradomics assay were compared to more conventional techniques (e.g., circular dichroism, fluorescence, and antigenicity). Finally, the bulk TTd was adsorbed to Al(OH)_3_ and AlPO_4_ to explore the potential of the assay on analyzing aluminum-adsorbed TTd. TTd vaccines are stable for years, but temperature exposure can accelerate loss of potency [18,19]. Exposure to 55 °C for three days has been reported to decrease TTd potency by 48%, whereas exposure to 53 °C for four days only decreased potency by 17%, indicating a turning point in TTd stability near these temperatures [20]. For our aberrant samples, we chose temperatures that would cover this range.

## 2. Materials and Methods 

Preparation of toxoids:Tetanus toxoids were obtained from three sources: In-house produced research grade TTd and GMP grade TTd from two manufacturers from the VAC2VAC consortium (Table 1). The toxoids were split in triplicates and thoroughly dialyzed (10-kDa MWCO, Thermo Scientific, Waltham, MA, USA) against 10 mM phosphate, pH 7.2 (obtained as 1 M solution from Sigma Aldrich, St. Louis, MO, USA). Subsequently, protein concentrations were determined by BCA and 5-mL samples at 100 µg/mL were then stored at various temperatures (Table 1). TTd-B1.3–TTd-B1.5 were not dialyzed prior to adsorption. The toxoids were adsorbed to Al(OH)_3_ (Brenntag, final concentration 1 mg/mL Al) and AlPO_4_ (Brenntag, final concentration 0.66 mg/mL Al) in presence of 50 mM phosphate, pH 7.2, to obtain a final TTd concentration of 20 Lf/mL. After two days of mixing at 4 °C, the adsorption efficiency was determined relative to the control samples. Subsequent temperature exposure was performed in accordance to Table 1. 

Enzymatic degradation and LC-MS analysis: two types of enzymatic degradation experiments were performed. In the first experiment, both the formation kinetics and identity of the TTd derived peptides were determined, followed by selecting three temperature sensitive peptides. The selection criteria for these peptides were: high overall peak area, low degrees of asparagine deamidation, and methionine oxidation, no peptide length variants of the same part of the sequence, and the peptides should have increasing intensities during the course of the digestion. In the second type of experiments the previously selected peptides were quantified after 20 h of digestion. Partial enzymatic digestions were carried out with 10 µg or 2 Lf TTd with 0.2 µg recombinant human cathepsin S (*E. coli*, activity: 80 U/µg, Merck) in the presence of 2 mM dithiothreitol, 2 mM ethylenediaminetetraacetic acid, and 100 mM sodium citrate, pH 5.0, at 37 °C. In the peptide selection and kinetics experiment, aliquots were diluted 20x in water containing 0.1 vol% formic acid with 1 fmol/µL angiotensin-III (Sigma Aldrich) after 0, 1, 4, 9, 26, and 32 h of incubation. When using stable isotope labeled (SIL) internal standards (Pepscan), at 20 h, 100 µL 0.1 vol% formic acid containing the following peptides was added: E-64 (Sigma Aldrich, protease inhibitor, 0.01 mM); EDNNITLK*, NLDRILR* and ASNWYFNHLK* at 25 fmol/µL; ASDWYFNHLK*, ASNWYFDHLK*, ASDWYFDHLK*, EDNDITLK*, EDDNITLK*, EDDDITLK* and DLDRILR* at 2.5 fmol/µL (K* contains 6 ^13^C and 2 ^15^N, R* contains 6 ^13^C and 4 ^15^N). Subsequently, the samples were purified by solid phase extraction (Sep-Pak C18, Waters) by washing with water containing 0.1 vol% formic acid (1 mL) followed by elution of the peptides with 600 µL 60 vol% acetonitrile containing 0.1% vol% formic acid, the eluent was dried and reconstituted in 15 µL DMSO followed by 285 µL 0.1 vol% formic acid. Samples were analyzed on a nanoLC-MS system on a Fusion Lumos Tribrid (ThermoFisher) as described elsewhere [17,21]. A 100-µm (I.D.) × 20-mm (L) trapping column packed with 5-µm Reprosil-Pur C18-AQ particles followed by a 50-µm (I.D.) × 31.5-cm (L) analytical column packed with 3-µm Reprosil-Pur C18-AQ particles were used for the separation of the peptides. The peptide mixtures were first trapped onto the trap column for 10 min at a flow rate of 5 µL/min (100% eluent A (0.1 vol% formic acid in water). The subsequent analytical separation was performed at a column flow rate of approximately 125 nL/min, achieved with a precolumn flow restrictor, all in conjunction with an Agilent 1290 Infinity HPLC system. A gradient of 10 to 40% eluent B (acetonitrile containing 0.1 vol% formic acid) in 10 min, followed by a 5-min gradient to 85% B was used for the analysis of peptides in conjunction with a SIL standard. For label-free quantification, a gradient of 10 to 35% B in 25 min followed by a 35 to 45% B in 10 min gradient was used. In-house-prepared gold/carbon-coated spray tips were used for electro-spray ionization. For the detection, an Orbitrap Fusion Lumos Tribrid mass spectrometer (Thermo Fisher, Waltham, MA, USA) was used with the following global settings for the label-free quantification: spray voltage 2100 V, Data Dependent Acquisition (DDA) with a maximum cycle time of 1.5 s between subsequent MS1 scans; MS1 scans were performed using the Orbitrap detector at a resolution of 30,000, maximum injection time of 50 ms and automatic gain control (AGC) target of 200,000. MS2 scans were performed in the Orbitrap detector at 15,000 resolution. For MS2 fragmentation, the included charge states were 1 to 8. Singly charged ions were only fragmented (by Collision Induced Dissociation (CID)) if their intensity was higher than 500,000 counts. Precursor ions with charge states of 2 to 4 were fragmented (CID) when the intensity was higher than 50,000 counts. Precursor ions with charge states 3 to 8 were also subjected to Electron Transfer Dissociation (ETD) if their intensities were higher than 200,000 counts. For experiments where SIL internal standard peptides were added, the following settings were adjusted to maximize MS1 performance: MS1 resolution 50,000, cycle time 1.0 s, MS2 detection in the ion trap, included charge states were 2 to 6 and only CID fragmentation was performed. Label free quantification and identification of peptides for the analysis of TTd-A1.1 was done with the QUAN module in PEAKS Studio X (Bioinformatics Solutions). Precursor mass tolerance was set to 5 ppm, fragment mass tolerance was set to 0.02 Da, enzyme was set to unspecific, variable modifications considered were: deamidation (NQ), oxidation (M) and potential formaldehyde adducts [22] (cyclic N terminus (+12), imine (+12) and methylol formation (+30.01), crosslinks between R and K (+24) and crosslinks between K and Y (+12)). A maximum of three variable PTMs were considered per peptide. No charge state filtering was applied to include the z = 1 peptides formed by cathepsin S digestion. The Quan module includes peptides with a false discovery rate of <1%. Peptide lists were exported and corrected for the relative abundance of the added angiotensin-III (Table S1). Quantification of the three selected peptides was achieved by comparing peak height in Xcalibur Quan (Thermo Fisher, Waltham, MA, USA), the deamidated forms were quantified by their total peak area to include iso-aspartate (Tables S2 and S3). 

Protein folding: tryptophan fluorescence was measured on a fluorescence spectrometer (LS55, PerkinElmer). One mL sample in a 1-cm quartz cuvette was measured with a high gain from 300 to 450 nm with a scan speed of 50 nm/minute and an excitation wavelength of 295 nm. Subsequently, Bis-ANS fluorescence was measured by addition of a 4,4′-Dianilino-1,1′-Binaphthyl-5,5′-Disulfonic Acid (Bis-ANS, Invitrogen, Carlsbad, CA, USA) solution to a final concentration of 10 µM to the non-adsorbed TTd. Emission fluorescence spectra were recorded after 20 min incubation at room temperature on a fluorescence spectrometer (LS55, PerkinElmer, Waltham, MA, USA) from 430 to 580 nm with a scan speed of 50 nm/minute and an excitation wavelength of 395 nm. Far-UV circular dichroism spectra (185 to 260 nm in steps of 0.5 nm) were recorded on a Chirascan circular dichroism spectrometer (Applied Photophysics, Leatherhead, United Kingdom). Measurements were performed on 400 µL TTd sample in a 1-mm quartz cuvette using a band width of 1 nm and a response time of 0.5 s at each wavelength point. Data of far-UV spectra were averaged over 10 subsequent scans per replicate. Higher order structure analysis was performed with qBiC (v1.1.2, Applied Photophysics, Leatherhead, United Kingdom) in accordance with the manufacturer’s recommendations. Note: samples were always compared to the 4 °C controls from the same source; Z-scores cannot be compared between different products.

Antibody binding: TTd binding to monoclonal antibody 10/134 (NIBSC) was measured on a Biacore T100 (GE Healthcare) similar to methods described elsewhere for diphtheria toxoids [23]. 0.1 mg/mL goat-anti-rat IgG UNLB (Southern Biotech) was immobilized on a CM5 chip (GE Healthcare) and used to subsequently capture mAb 10/134 at a flow of 10 µL/min for 5 min. Maximum binding of the TTd was measured after a 5 min flow of 5 µL/min. Responses were normalized relative to the 4 °C controls. 

Monomer quantification. The TTd monomer content was determined by size exclusion chromatography. Samples were centrifuged for 60 min at 17,000 g to remove large aggregates prior to analysis. Four µL of the supernatant was injected onto a ACQUITY UPLC Protein BEH SEC Column (200A, 1.7 µm, 4.6 mm × 300 mm, 10k–500k, Waters) with phosphate buffered saline (Gibco) at a 0.3 mL/min flowrate for 18 min. Detection was performed by using UV absorbance at 215 nm.

## 3. Results and Discussion

### 3.1. Preparation of Aberrant Toxoids

Development of stability indicating tests and batch-to-batch consistency assays for biologicals requires known aberrant products, which the test should be able to distinguish from high quality products. Acquiring real toxoids intended for human (or veterinary) use that did not meet current quality control (QC) criteria was not feasible because manufacturers with a robust production process generate no failed batches and any potential manufacturers that struggle with consistency would be unlikely to cooperate in studies such as these. As such, the best option is to alter TTd drug products in such a way that they should fail the current QC criteria. Previous studies have shown that tetanus toxoid rapidly loses potency upon exposure to 55 °C for three days [20]. Therefore, an accelerated degradation protocol was implemented whereby the TTd samples were exposed to temperatures covering this range as a means to obtain aberrant samples. In-house produced non-adsorbed tetanus toxoid (TTd-A1.1) was used to conduct an initial evaluation of our degradomics assay concept. The toxoid was subjected to elevated temperatures: 37 °C for 30 days and 50, 55, 60, 65 °C for two days. Afterwards, the toxoids (TTd-A1.1) were stored at 4 °C for several months prior to analysis. During this time period, visible aggregates formed in the toxoids that were exposed to the three highest temperatures, where the severity of the aggregation increased with increasing temperature. For the follow-up experiments (comparison of batches from different manufacturers and comparison of various batches of one manufacturer) samples were exposed to all temperatures for two days, with subsequent storage at 4 °C and follow-up analysis within a month. During this time, no visible aggregates were formed. It is likely that the damage to the protein folding triggered further accelerated aggregation. Although this results in differences between the samples used in the initial screening and those used in the final assay, the alterations could be picked up for both TTd-A1.1 and the follow-up experiments. 

### 3.2. Degradomics Analysis

**Figure 1:**
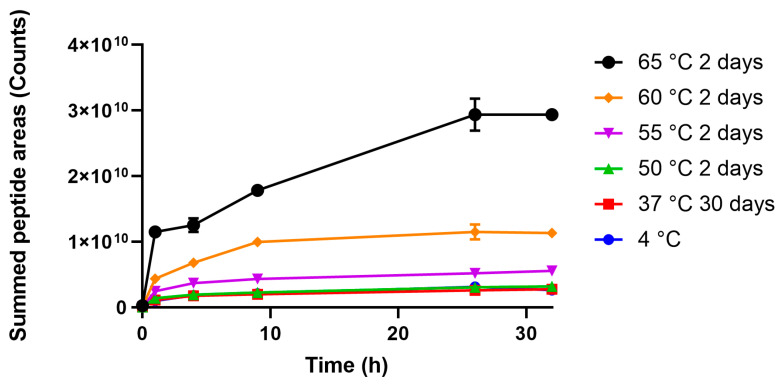
Summed areas of peptides formed during partial digestion of temperature exposed TTd-A1.1 by cathepsin S. Inset: zoomed graph to allow comparison between TTd exposed to the lower temperature ranges. Peptides were quantified by label-free quantification. Error bars represent SD of three measurements.

Temperature exposed TTd-A1.1 was subjected to cathepsin S digestion and the formation of peptides was monitored by nanoscale LC-MS. These peptides (not modified by formaldehyde) were identified and quantified by peak area with PEAKS Studio X. To reduce inter-measurement variation, the peak areas were corrected by addition of a fixed amount of angiotensin-III as an internal standard. In total, 396 tetanus toxin-derived peptides were identified and used for the initial comparison. In this comparison, the total peptide intensity was summed per time point (Figure 1). At exposure to 55 °C and above, the overall area of the peptides increased dramatically. While this initial approach gave insight into the overall reaction kinetics, degradation reactions in various parts of the protein are not detected and potential opposite effects of exposure to elevated temperature could cancel out.

**Figure 2:**
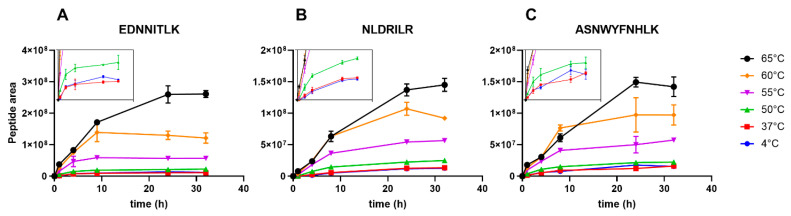
Formation of peptides (**A**) EDNNITLK, (**B**) NLDRILR, and (**C**) ASNWYFNHLK over time obtained from cathepsin S-mediated digestion of TTd-A1.1 after exposure to various temperatures (2 (50, 55, 60, 65 °C) or 30 days (37 °C)). Peptides were quantified by label-free quantification. Insets: zoomed graphs show the differences between exposure to 4 and 37 °C, and 50 °C. Error bars represent SD of three measurements.

To further improve the sensitivity of the assay and to find specific areas of the protein that underwent thermal degradation, peptides were sorted according to their 50 °C to 4 °C ratio measured at the 4 h time point. Further selection of the peptides was based on the overall peak area to include the most abundant peptides. Peptides with high degrees of asparagine deamidation and methionine oxidation were excluded, as well as peptide length variants of the same part of the sequence. The final criterion was that the peptides should have increasing intensities during the course of the digestion. The reason is that some peptides are cleaved for a second time during digestion, reducing the amount of the precursor peptide and complicating the quantification. The three peptides that complied with our criteria were E^1066^DNNITLK^1073^, N^1220^LDRILR^1226^, and A^1286^SNWYFNHLK^1295^ (Figure 2). These peptides all reside in the tetanus toxoid heavy chain (Figure S1). NLDRILR and ASNWYFNHLK are in very close proximity and part of the same β-sheet, which explains their similar sensitivity to temperature exposure; apparently this part of the molecule is easily affected by temperature exposure. Peptide EDNNITLK is part of an unrelated large β-sheet in another part of the molecule. To further optimize analysis time and assay performance, synthetic stable isotope labeled (SIL) peptides were used as internal standard for more robust, absolute, quantification and instead of plotting the kinetics, the amount of peptide at a set time point (20 h) was analyzed and compared (a representative chromatogram is depicted in Figure S2). Addition of SIL internal standards for these peptides eliminates most of the inter-assay variation, and most likely avoids the need for expensive high-resolution mass spectrometers and allows further transfer of the method to more conventional liquid chromatography systems. Because of the relatively high abundance of asparagine residues in tetanus toxin (9.21%), it was not possible to select peptides without asparagine residues and completely avoid deamidation. To overcome this drawback, SIL internal standard peptides with aspartate residues instead of asparagine residues were used. Because deamidation results in a mixture of aspartate and *iso*-aspartate [24], the peak area was used to quantify all deamidated forms (which are not fully resolved) instead of quantifying by peak height (useful to compare the same MS1 scan of coeluting peptides). Determining the exact degree of deamidation by degradomics analysis is difficult, because this would require complete digestion of the protein by a selective protease. However, our current analysis is sufficient to demonstrate that the selected peptides were not deamidated below the concentrations obtained with the 4 °C reference sample.

**Figure 3:**
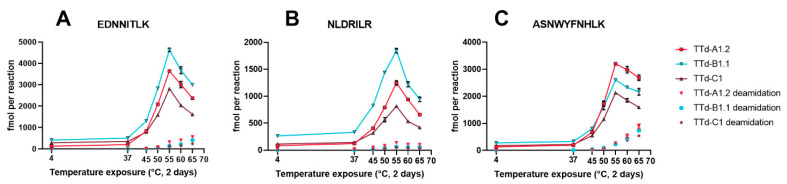
Absolute quantification of (**A**) EDNNITLK, (**B**) NLDRILR, and (**C**) ASNWYFNHLK formed by partial digestion (at t = 20 h) with cathepsin S of tetanus toxoids (TTd-A1.2, TTd-B1.1 and TTd-C1) that were pre-exposed to elevated temperatures. Connected points indicate the sum of the native peptides and their deamidated forms, single points represent the deamidated forms only. Error bars represent the SD of three temperature exposed aliquots of the same TTd batch.

Absolute quantification of the peptides obtained after 20 h of enzymatic degradation was then applied to heat-treated bulk tetanus toxoids from two manufacturers to confirm that our new degradomics-based assay can be utilized to detect aberrant toxoids regardless of their source. The toxoids from both manufacturers and the reference toxoid showed an increased enzyme-mediated formation of the selected peptides after exposure to 45 °C for two days compared to their 4 °C counterparts (Figure 3). As expected, small differences in the absolute concentrations of formed peptide were observed between the toxoids from different sources but the overall trends were remarkably similar. Identical protein concentrations were used, so the differences most likely originate from dissimilarities in protein composition and purity. Unexpectedly, exposure to 60 °C and 65 °C decreased the amount of peptide present compared to 55 °C, although the concentrations were still well above those of the 4 °C controls. This decrease was most prominent for EDNNITLK and NLDRILR. Our initial screening experiments (which used TTd-A1.1 with increasing amounts of visible aggregates at the highest temperatures), did not show this trend. A probable explanation is that certain intermediate unfolded states of the protein are slightly more resistant to enzymatic proteolysis than those predominantly formed upon exposure up to 55 °C, and further degradation and the formation of visible aggregates could result in faster enzymatic proteolysis. The absolute amount of deamidated peptides observed was proportional to the increase in temperature exposure, but does not explain the decrease in intensity at the highest temperatures. Deamidation of EDNNITLK and NLDRILR was limited (maxima at 65 °C between 13–24 and 6–16%, respectively), however, exposure to 65 °C resulted in 33 to 34% deamidation of ASNWYFNHLK, although care should be taken with interpretation of this data; the enzymatic degradation is not complete and not selective, which are requirements for accurate determination of the extent of asparagine deamidation. Overall quantification of E^1066^DNNITLK^1073^, N^1220^LDRILR^1226^, and A^1286^SNWYFNHLK^1295^ released from partial cathepsin S digestion could easily distinguish non-adsorbed tetanus toxoids stored at 4 °C from those exposed to 45 °C and higher, for both our research toxoid and toxoids provided by two manufacturers of vaccines. 

**Figure 4:**
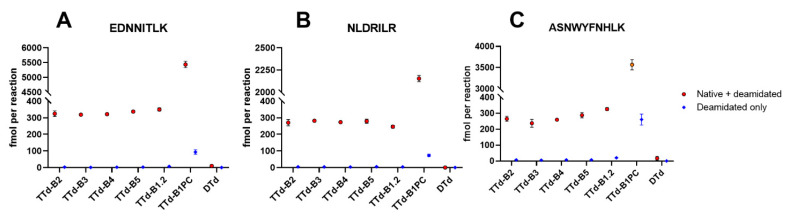
Temperature sensitive formation of peptides (**A**) EDNNITLK, (**B**) NLDRILR, and (**C**) ASNWYFNHLK by partial digestion with cathepsin S of tetanus toxoid batches of the same manufacturer. TTd-B1.2 was used as a reference and is two years older than TTd-B2–TTd-B5, an aliquot of TTd-B1.2 exposed to 55 °C for two days is used as a positive control (TTd-B1PC). Diphtheria toxoid of the same manufacturer was used as a negative control. Error bars represent the SD of three enzymatic digestions of aliquots of the same batch.

To evaluate the potential of this new assay for batch-to-batch consistency testing, four additional batches from manufacturer B were analyzed. As such, a reference sample (TTd-B1.2 untreated, stored at 4 °C) and an aberrant sample (TTd-B1PC stored at 55 °C for two days) were prepared from TTd-B1. The concentrations of the enzymatically released peptides were very similar among the different batches (Figure 4) and the aberrant sample can easily be distinguished from the toxoids stored at 4 °C. Acceptable limits should be set by manufacturers for their specific products, but overall these results show that the assay is suitable for batch-to-batch consistency testing. 

### 3.3. Evaluation of Heat-Treated Toxoids by Traditional Techniques

In order to put the temperature-induced alterations to the tetanus toxoids observed with the degradomics analysis into perspective, the toxoids were also analyzed by more traditional techniques. The parameters studied included: antigen binding (biosensor analysis), protein folding (circular dichroism, tryptophan fluorescence and Bis-ANS fluorescence), and aggregation (size-exclusion chromatography). 

**Figure 5:**
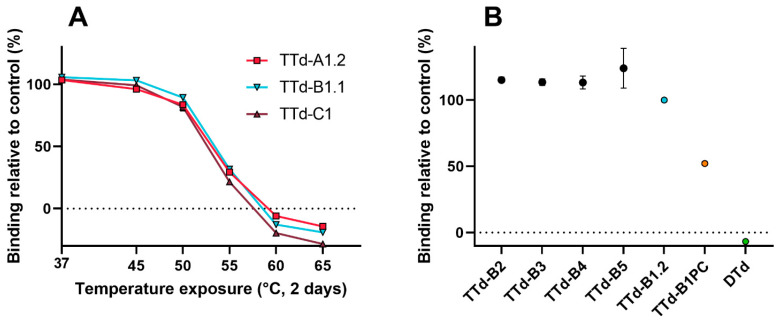
Biosensor analysis of tetanus toxoids. (**A**) Decrease in antibody binding relative to the 4 °C control. (**B**) Comparison of antibody binding between the various batches relative to TTd-B1.2. Error bars represent the SD of three aliquots of the same batch.

The decrease in antibody binding of TTd showed similar trends for the batches of all three manufacturers: a moderate decrease in antibody binding compared to the 4 °C control was observed after exposure to 50 °C (10–18% reduction), the epitope was severely affected upon exposure to 55 °C for two days (68–79% reduction), and exposure to higher temperatures completely destroyed the epitope (Figure 5A). 

**Figure 6:**
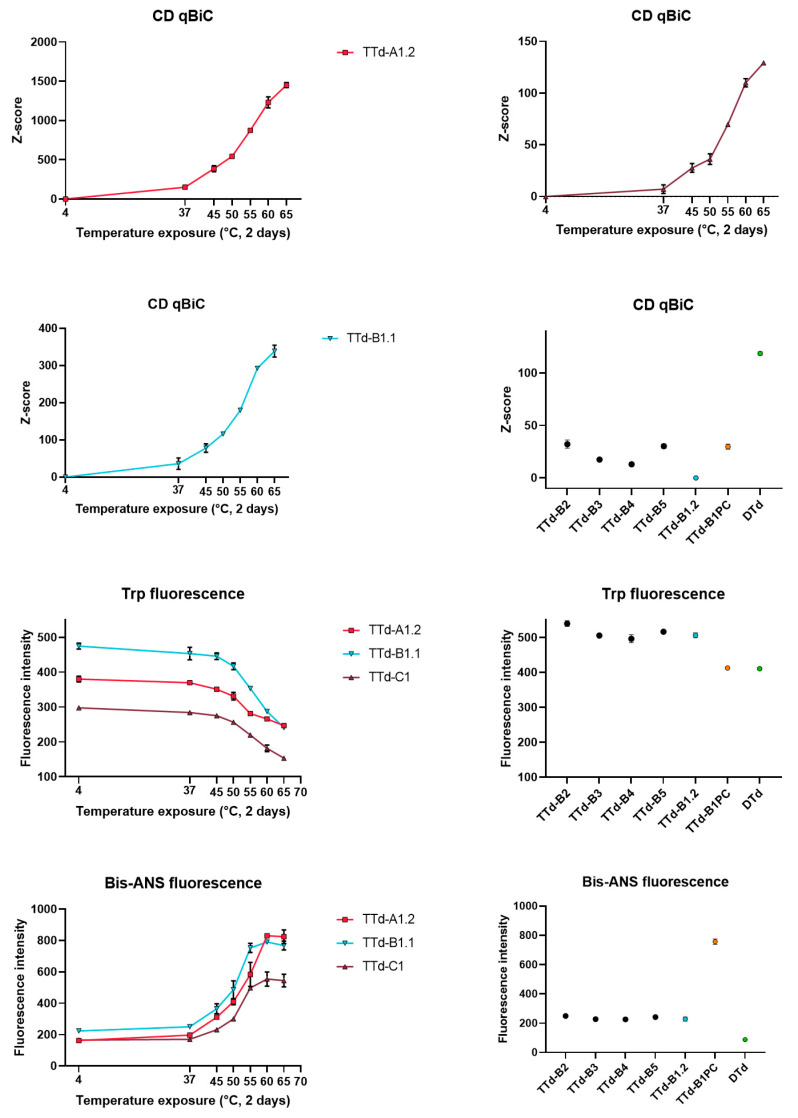
Evaluation of protein folding by circular dichroism (CD), tryptophan fluorescence and Bis-ANS fluorescence. CD spectra were analyzed using qBiC to compare differences in higher order structure. Error bars represent the SD of three aliquots of the same batch.

Protein folding analysis by circular dichroism (CD), tryptophan fluorescence and Bis-ANS fluorescence all showed temperature dependent alterations of the protein structure (Figure 6). CD spectra were compared using qBiC, a software package intended for protein higher order structure (HOS) analysis. This analysis was very sensitive and could already pick up differences between the 4 °C reference and the toxoids that were exposed to 37 °C for two days, subjecting the toxoids to higher temperatures resulted in larger spectral differences. Moreover, differences between the four new batches and the older reference batch could also be detected. Analysis of the toxoids by tryptophan and Bis-ANS fluorescence also showed differences after exposure to 45 °C.

**Figure 7:**
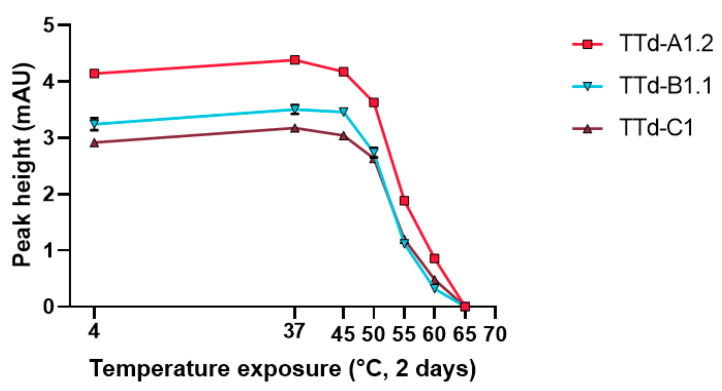
Monomer quantification of temperature exposed tetanus toxoids by size exclusion chromatography. Error bars represent the SD of three aliquots of the same batch.

Finally, protein aggregation was inferred from size exclusion chromatography. Exposure to temperatures above 50 °C rapidly decreased the monomer content (with negligible effects on total protein concentration as determined by BCA prior to pre-SEC centrifugation) (Figure 7 and Figure S3). This is consistent with the trend observed with all other techniques.

Although our degradomics analysis and most other techniques picked up differences to the toxoids after exposure to 45 °C for two days, the epitope where mAb 10/134 binds was still mostly intact after exposure to 50 °C, where loss of the epitope started at exposure to 55 °C. While this is only one epitope, it is consistent with known decreases in in vivo potency found by others [20]. Despite our degradomics assay being very sensitive and picking up changes before potency of the toxoid is completely lost, the superior performance of the degradomics assay compared to the animal tests should avoid rejection of good batches.

### 3.4. Al(OH)_3_ and AlPO_4_ Adsorbed Tetanus Toxoid

**Figure 8:**
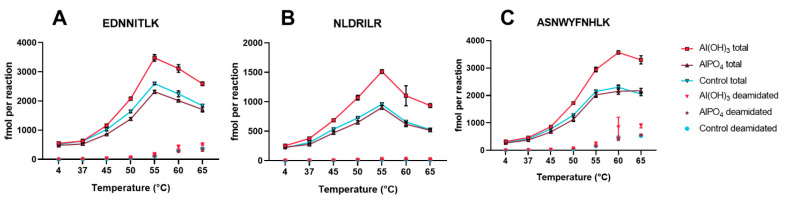
Temperature sensitive formation of peptides (**A**) EDNNITLK, (**B**) NLDRILR, and (**C**) ASNWYFNHLK by cathepsin S from Al(OH)_3_ and AlPO_4_ adsorbed tetanus toxoids. Connected lines show the sum of the native and the deamidated peptides, single points indicate the sum of all variants of the deamidated peptides. Error bars represent SD of three individually adsorbed samples.

Tetanus toxoids are usually adsorbed to Al(OH)_3_, AlPO_4_ or a mixture of the two. Analysis of antigens adsorbed to aluminum-based adjuvants is challenging with many traditional techniques, because the samples are highly turbid, the proteins are not completely in solution and several vaccines contain multiple antigens. Although showing batch consistency through testing of the drug substance (before adsorption) could contribute to the replacement of in vivo tests of the drug product, an assay that could be directly applied to the drug product would be highly favorable. To evaluate the feasibility of utilizing our assay under these conditions, TTd of manufacturer B was adsorbed to Al(OH)_3_ and AlPO_4_ separately, to achieve adsorbed tetanus toxoids with 10 Lf/mL TTd and 1 mg/mL or 0.66 mg/mL Al^3+^, respectively. Along with a control (10 Lf/mL TTd without aluminum-based adjuvant), these samples were mixed overnight and adsorption was determined by relative decrease in protein concentration of the supernatant compared to the control. Adsorption of Al(OH)_3_ was more effective (48%, SD 2%) than AlPO_4_ (8%, SD 1%). Subsequently, the samples were split and the aliquots were subjected to elevated temperatures for two days and then analyzed with our degradomics assay. Exposure of the AlPO_4_-adsorbed TTd resulted in similar kinetic profiles for peptides ASNWYFNHLK and NLDRILR, whereas EDNNITLK was slightly more sensitive to temperature exposure when the TTd was combined with AlPO_4_ (Figure 8). Al(OH)_3_ adsorbed TTd was more sensitive to temperature exposure for all three peptides, even though the 4 °C starting points were very similar, indicating that adsorption itself did not alter the degradation kinetics of these peptides. The increased temperature sensitivity observed with TTd adsorbed to Al(OH)_3_ compared to the control and the AlPO_4_ adsorbed TTd is likely due to the higher percentage of adsorption, where the adsorbed product’s structure is altered in such a way that it is less stable. Overall, the potential for drug product testing with the enzymatic degradation assay is a clear advantage over many other in vitro assays, which are usually limited to analysis of drug substance.

In general, the degradomics assay does not require a biological reference for every experiment, but a number of synthetic peptides, which can be well defined, quantified, and are readily available. This is a major advantage compared to other biochemical techniques which would require antibodies or toxoids. Although the activity of the recombinant cathepsin S is critical, activity tests with fluorescent probes offer an easy method for quality control of the enzyme [25].

## 4. Conclusions

A new type of degradomics-based assay was developed for the analysis of aluminum-adsorbed and non-adsorbed tetanus toxoids, which could be applicable to other protein-antigens as well. By analyzing the formation of peptides from treatment of tetanus toxoid by recombinant human cathepsin S, an important enzyme involved in antigen processing in vivo, aberrant tetanus toxoids could be distinguished from control toxoids. Overall, with some further optimization, implementation and validation in a QC environment, the assay may have potential for use in both stability and batch-to-batch consistency studies as an alternative for in vivo potency studies.

## Figures and Tables

**Table 1:** Overview of the tetanus toxoid batches used in this study.

Name	Source	Experiment	Temperature Exposure (Duration)
TTd-A1.1	In-house	Peptide selection and evaluation of kinetics	4, 50, 55, 60, 65 °C, (2 days) 37 °C, (30 days)
TTd-A1.2	In-house	Quantification with isotopically labeled standard	4, 37, 45, 50, 55, 60, 65 °C, (2 days)
TTd-B1.1	Manufacturer B	Quantification with isotopically labeled standard	4, 37, 45, 50, 55, 60, 65 °C, (2 days)
TTd-C1	Manufacturer C	Quantification with isotopically labeled standard	4, 37, 45, 50, 55, 60, 65 °C, (2 days)
TTd-B1.2	Manufacturer B	Evaluation of batch consistency	4 °C
TTd-B1PC	Manufacturer B	Evaluation of batch consistency positive control	55 °C, (2 days)
TTd-B2	Manufacturer B	Evaluation of batch consistency	4 °C
TTd-B3	Manufacturer B	Evaluation of batch consistency	4 °C
TTd-B4	Manufacturer B	Evaluation of batch consistency	4 °C
TTd-B5	Manufacturer B	Evaluation of batch consistency	4 °C
DTd	Manufacturer B Diphtheria toxoid	Evaluation of batch consistency negative control	4 °C
TTd-B1.3	Manufacturer B	Adsorbed toxoids control (no adjuvant added)	4, 37, 45, 50, 55, 60, 65 °C, (2 days)
TTd-B1.4	Manufacturer B	Adsorbed toxoids Al(OH)_3_ adsorbed	4, 37, 45, 50, 55, 60, 65 °C, (2 days)
TTd-B1.5	Manufacturer B	Adsorbed toxoids AlPO_4_ adsorbed	4, 37, 45, 50, 55, 60, 65 °C, (2 days)

## Supplementary Materials

The following are available online at https://www.mdpi.com/2076-393X/8/4/712/s1, Figure S1: Crystal structure of tetanus toxin where the selected peptides that were formed faster from cathepsin S digestion after temperature exposure are highlighted, Figure S2: Representative chromatogram and spectra of the tetanus toxoid degradomics analysis., Figure S3: Representative size exclusion chromatography chromatogram of TTd-B1.1 after exposure to 4, 37, 45, 50, 55, 60, or 65 °C for two days. Table S1 contains the exported PEAKS data used for Figure 1 and Figure 2. Table S2 contains the quantification data used for Figure 3 and Figure 4. Table S3 contains the quantification data used for Figure 8. The data presented in this study are openly available in the MassIVE mass spectrometry repository at https://doi.org/doi:10.25345/C58F6R.
