# Supplementary material for: Degradomics-Based Analysis of Tetanus Toxoids as a Quality Control Assay
Source: Vaccines (Basel). 2020 Dec 1;8(4):712. doi: 10.3390/vaccines8040712 (PMC7712181; doi:10.3390/vaccines8040712)
Supplement: Supplementary file 1 [file vaccines-08-00712-s001.zip › 20201119_supplementalfiguresTTddegradomics_rebuttal.docx]

Figure S1. Crystal structure of tetanus toxin where the selected peptides that were formed faster from cathepsin S digestion after temperature exposure are highlighted. A fragment = pink, B fragment = grey, EDNNITLK = green, NLDRILR = blue, ASNWYFNHLK = red. PDB 5N0B and Pymol (version 2.1.1, DeLano Scientific) were used to generate the figure.

Figure S2. Representative chromatogram and spectra of the tetanus toxoid degradomics analysis. The tetanus toxoid used for this chromatogram and these spectra is TTd-A1.2, exposed to 65 °C for 2 days prior to cathepsin S digestion. Panel A shows the base peak (top) chromatogram and extracted ion chromatograms (5 ppm) of peptides ASNWYFNHLK, NLDRILR and EDNNITLK. The extracted ion chromatogram for EDNNITLK also shows another peptide (DLGGELCIK), eluting at a different time. This does not influence the analysis. Panel B shows a MS1 spectrum of EDNNITLK and the internal standard EDNNITLK*. Panel C shows the MS2 fragmentation of EDNNITLK (top) and EDNNITLK* (bottom).

Figure S3. Representative size exclusion chromatography chromatogram of TTd-B1.1 after exposure to 4, 37, 45, 50, 55, 60 or 65 °C for 2 days. Large particulates were removed by centrifugation prior to injection. A reference mixture had the following retention times: uracil 14.5 min, RNAse A 11.8 min, ovalbumin monomer 9.8 min, ovalbumin dimer 8.7 min, thyroglobulin 6.6 min.
